# Reduced *ech-6* expression attenuates fat-induced lifespan shortening in *C. elegans*

**DOI:** 10.1038/s41598-022-07397-9

**Published:** 2022-03-01

**Authors:** Yasmine J. Liu, Arwen W. Gao, Reuben L. Smith, Georges E. Janssens, Daan M. Panneman, Aldo Jongejan, Michel van Weeghel, Frédéric M. Vaz, Melissa J. Silvestrini, Louis R. Lapierre, Alyson W. MacInnes, Riekelt H. Houtkooper

**Affiliations:** 1 Laboratory Genetic Metabolic Diseases, Amsterdam Gastroenterology, Endocrinology, and Metabolism, Amsterdam Cardiovascular Sciences, Amsterdam UMC, University of Amsterdam, Meibergdreef 9, 1105 AZ Amsterdam, The Netherlands; 2 Bioinformatics Laboratory, Department of Epidemiology and Data Science, Amsterdam UMC, University of Amsterdam, Meibergdreef 9, AZ, Amsterdam, The Netherlands; 3 Core Facility Metabolomics, Amsterdam University Medical Centers, University of Amsterdam, Amsterdam, The Netherlands; 4 Department of Molecular Biology, Cell Biology and Biochemistry, Brown University, Providence, RI 02912 USA; 5 Present Address: Laboratory of Integrative Systems Physiology, Institute of Bioengineering, École Polytechnique Fédérale de Lausanne, 1015 Lausanne, Switzerland; 6 Present Address: Radboud Center for Mitochondrial Medicine, Department of Pediatrics, Amalia Children’s Hospital, Nijmegen, The Netherlands

**Keywords:** Molecular biology, Proteolysis, RNAi, Transcription, Cell biology, Autophagy, Cell signalling, Organelles, Proteolysis, Genetics, Gene expression, RNAi

## Abstract

Deregulated energy homeostasis represents a hallmark of aging and results from complex gene-by-environment interactions. Here, we discovered that reducing the expression of the gene *ech-6* encoding enoyl-CoA hydratase remitted fat diet-induced deleterious effects on lifespan in *Caenorhabditis elegans*, while a basal expression of *ech-6* was important for survival under normal dietary conditions. Lipidomics revealed that supplementation of fat in *ech-6*-silenced worms had marginal effects on lipid profiles, suggesting an alternative fat utilization for energy production. Transcriptomics further suggest a causal relation between the lysosomal pathway, energy production, and the longevity effect conferred by the interaction between *ech-6* and fat diets. Indeed, enhancing energy production from endogenous fat by overexpressing lysosomal lipase *lipl-4* recapitulated the lifespan effects of fat diets on *ech-6*-silenced worms. Collectively, these results suggest that the gene *ech-6* is potential modulator of metabolic flexibility and may be a target for promoting metabolic health and longevity.

## Introduction

Lipids are an essential energy source required for numerous biological processes and tightly modulated to maintain organismal health^1,2^. Dysfunctional lipid metabolism is associated with serious health problems including obesity, type 2 diabetes mellitus and cardiovascular diseases^1,3,4^. These pathological conditions have become epidemics of alarming proportions in many western countries where diets are enriched in fat^5,6^. In addition, a growing body of evidence underscores the association between abnormal lipid metabolism and the aging process, in which a deficiency of fat breakdown occurs during aging and leads to fat accumulation in aged organisms^7,8^. Preventing fat accumulation, for instance by intermittent fasting, ameliorates aging and postpones the advent of aging-related metabolic disorders^9^.

Metabolic flexibility refers to a state of efficient switching between metabolic pathways^2^. A flexible metabolism allows cells to adapt to fuel usage and to switch efficiently between nutrient sources depending on the environmental conditions^2^. Gradual loss of this process during aging is a causative factor for increased susceptibility to aging-related metabolic disorders, yet the incidence and severity of these complex diseases vary considerably^10,11^. The underlying causes of the variability involve not only discrete genetic and environmental factors, but also the interactions between the two^11,12^. Gene-by-environment interactions (GxE) imply genetic predispositions that are differentially expressed depending on the environment and a genetic contribution to a specific phenotypic outcome that can be estimated when the environment is stable^11,12^. Previous forward and reverse genetic studies in model organisms have unraveled a number of susceptibility and resistance alleles that are relevant to complex traits and specific diseases^13–15^. However, our knowledge about the GxE pairs that explain the variability in the degree of metabolic flexibility and the rate of aging is still limited.

*Caenorhabditis elegans* is one of the most widely used model organisms in the aging field^16,17^. Its capability to respond to various dietary interventions facilitate the identification of GxE interactions influencing complex traits and the mechanistic delineation of molecular pathways^18–22^. Particularly, many of the core metabolic pathways that modulate aging in mammals are conserved in *C. elegans*^23,24^. For example, emerging evidence shows that lipid metabolism influences the lifespan of model organisms including *C. elegans*^25^ and depletion of regulators in fat metabolism shortens the lifespan of *C. elegans*^26,27^. Nevertheless, the interaction between fat metabolism and aging is more convoluted, as different classes of long-lived mutant worms exhibit distinct levels of lipid content depending on the longevity signaling pathways involved, such as the insulin/insulin-like growth factor (IGF-1) pathway, the mTOR pathway, and the AMPK pathway^8,28–30^.

In this study we aim to elucidate how genes and dietary fat converge at the level of metabolic flexibility to affect longevity. We report that *ech-6*, an enoyl-CoA hydratase, modulates worm lifespan in response to a dietary excess of fat. *ech-6* encodes an enoyl-CoA hydratase and is proposed to act in branched-chain amino acid catabolism^31^, for example, by catalyzing methacrylyl-CoA to 3-hydroxyisobutyryl-CoA in the valine catabolic pathways^32^. We show that supplementation of dietary fat P-80 in wild-type animals leads to lifespan shortening, possibly as a result of metabolic inflexibility induced by energy overload. Knockdown of *ech-6* under normal dietary conditions shortens lifespan, possibly caused by cellular energy crisis through suppressed mitochondrial function and compromised amino acid catabolism. Interestingly, knockdown of *ech-6* in combination with P-80 supplementation protects animals against both fat diet- and *ech-6* RNAi-induced detrimental effect on lifespan. The underlying mechanism that accounts for the lifespan effect lies in the upregulated energy production and lysosome-related processes. The findings of this study indicate that the gene *ech-6* represents a factor, with the potential to be used to fine-tune the degree of metabolic flexibility in response to excessive dietary fat intake to modulate lifespan.

## Results

### Reduced expression of *ech-6* prevents accelerated aging caused by a dietary excess of fat

**Figure 1:**
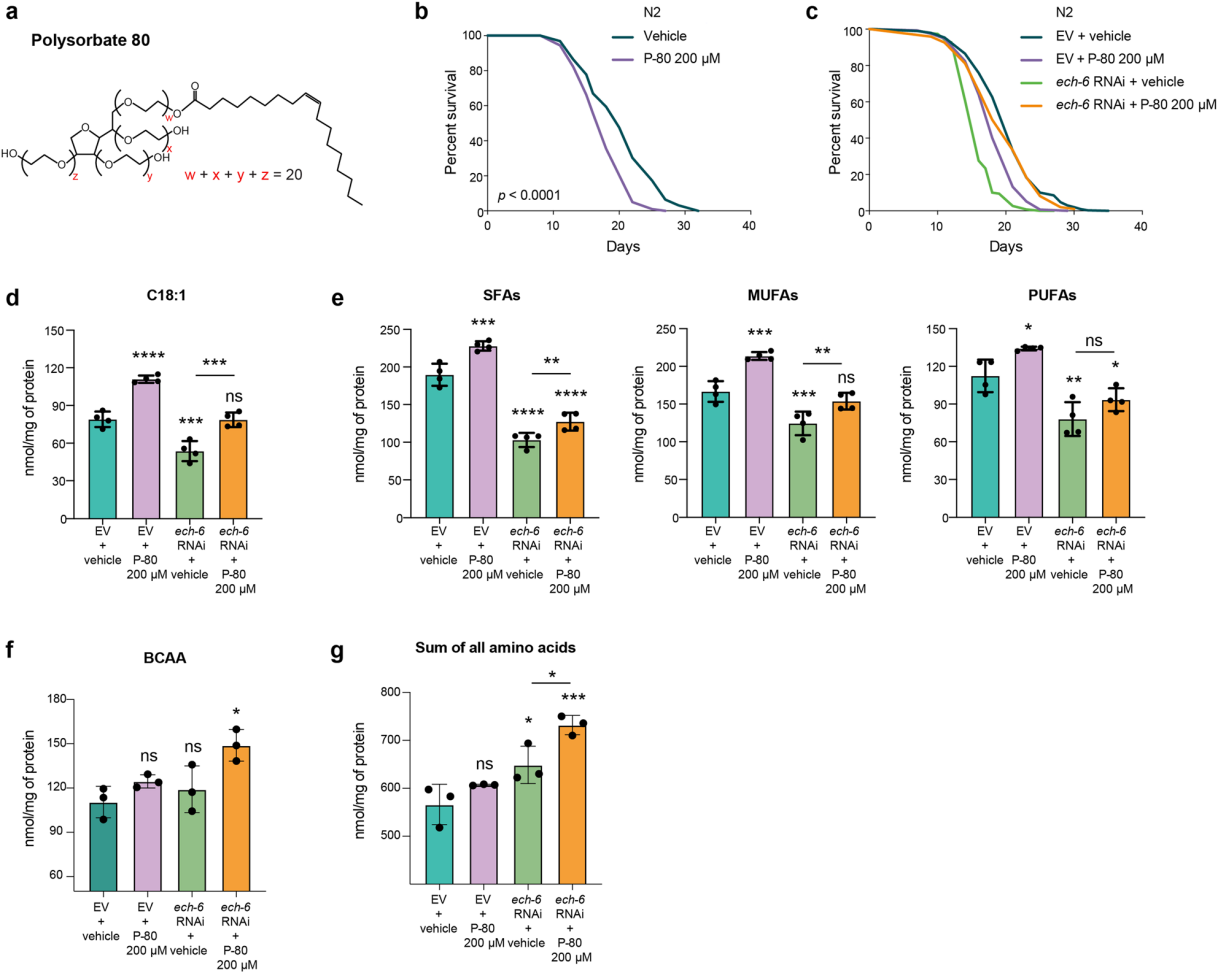
Knockdown of *ech-6* antagonizes the effects of a fat diet on lifespan, oleic acid content and fatty acid saturation profile. (**a**) Chemical structure of polysorbate 80 (P-80). P-80 consists of a hydrophilic group of ethylene oxide polymers and oleic acid. (**b**) Survival curves of worms fed with 200 µM P-80 showing that worms live significantly shorter when subjected to a dietary excess of fat (*p* < 0.0001, log-rank test). Vehicle: H_2_O, which applies to all the following figures. (**c**) Survival curves of worms treated with *ech-6* RNAi or P-80 supplementation. Knockdown of *ech-6* protects worms against P-80 diet-induced lifespan decrease (*p* < 0.0001, log-rank test), while shortening lifespan under regular NGM dietary conditions (*p* < 0.0001, log-rank test). (**d**) Mass spectrometry (MS) quantification of oleic acid in worms exposed to the P-80 diet. P-80 supplementation increases the level of oleic acid in empty vector-treated wild-type (N2) worms while compensates for the reduced level caused by reduction of *ech-6* by RNAi. Mean ± SD of 4 biological replicates. (**e**) MS quantification of saturated fatty acids (SFAs), monounsaturated fatty acids (MUFAs), and poly unsaturated fatty acids (PFAs). Mean ± SD of 4 biological replicates. (**f**) UPLC-MS/MS quantification of branched-chain amino acids (BCAA). Knockdown of *ech-6* in combination with P-80 supplementation significantly increases the level of BCAA. Mean ± SD of 3 biological replicates. (**g**) Quantification of total amino acids. Knockdown of *ech-6* significantly increases the level of total amino acids. Mean ± SD of 3 biological replicates. *p < 0.05; **p < 0.01; ***p < 0.001; ****p < 0.0001; ns, not significant; one-way ANOVA, Holm-Sidak correction. An asterisk directly above a box refers to statistical significance compared to empty vector (EV)-treated wild-type (N2) worms fed a regular NGM diet, while an asterisk over a line indicates statistical significance compared to inhibition of *ech-6* by RNAi. See also Table S1 for lifespan data.

To study the impact of dietary interventions on worm lifespan, particularly a fat diet intervention, we cultured wild-type N2 worms on plates containing soluble fat polysorbate 80 (P-80). We selected this compound, since (1) it contains a long-chain fatty acid, oleic acid, one of the important constituent fatty acids in adult *C. elegans*^8,26,33^, and (2) it allows for uniform solubilization in the culture plates (Fig. 1a). We found that worms exposed to P-80 supplementation had a reduced lifespan (Fig. 1b; Supplementary Table S1), in line with the lifespan effect of excess dietary fat in mice^34^. Next, we conducted an RNAi-based small-scale lifespan screen to search for metabolic genes that could alter the susceptibility to the detrimental effects of this fat diet. Our initial screen prioritized the genes involved in mitochondrial transport and fatty acid β-oxidation, including *dif-1*, *cpt-2*, *acdh-7*, *acdh-12*, and *ech-6.* The most prominent candidate that emerged was *ech-6* (enoyl-CoA hydratase 6; orthologue of mammalian *ECHS1*). Knockdown of *ech-6* protected worms against the lifespan shortening caused by P-80 supplementation (Fig. 1c; Supplementary Table S1). In addition, depletion of *ech-6* shortened lifespan (Fig. 1c; Supplementary Table S1), as previously reported^35^, suggesting the requirement of a basal expression of *ech-6* for a normal lifespan. Taken together, these results suggest that *ech-6* interacts with fat diets to influence the rate of aging.

As oleic acid is a major component of P-80, we then asked whether feeding worms the fat diet would alter the endogenous level of oleic acid. To test this, we analyzed the fatty acid profile of worms exposed to the P-80 diet using a targeted metabolomics platform^8^. Supplementation of P-80 to wild-type worms elevated the level of oleic acid (Fig. 1d). Conversely, knockdown of *ech-6* decreased the level of oleic acid, which was increased to near-normal levels with the addition of P-80 (Fig. 1d). As the saturation level of fatty acids was shown to be involved in longevity regulation in *C. elegans*^36,37^, we next determined the effects of the P-80 diet on the degree of fatty acid saturation. Feeding wild-type worms with the P-80 diet significantly increased the levels of all three types of fatty acids including saturated fatty acids (SFAs), mono-unsaturated fatty acids (MUFAs) and poly-unsaturated fatty acids (PUFAs) (Fig. 1e; Supplementary Table S5). Conversely, knockdown of *ech-6* significantly decreased the levels of SFAs, MUFAs, and PUFAs (Fig. 1e). Supplementing *ech-6*-silenced worms with P-80 specifically increased both SFAs and MUFAs, while having no effect on PUFAs (Fig. 1e). These data show that depletion of *ech-6* and supplementation of P-80 exhibit reciprocal effects on the level of oleic acid and the saturation state of fatty acids.

As the gene *ech-6* has been annotated as an enoyl-CoA hydratase involved in the branched-chain amino acids (BCAA) breakdown which plays an important role in energy production^32,35^, we determined the effects of reduced *ech-6* expression on amino acid profiles using ultra performance liquid chromatography tandem-mass spectrometry (UPLC-MS/MS)^8^. Knockdown of *ech-6* did not change the level of BCAA, but significantly elevated the overall levels of amino acids of which increased alanine made up a major portion (Fig. 1f,g and Supplementary Fig. S1). Interestingly, we observed an increased level of BCAA when supplementing *ech-6*-silenced worms with P-80, whereas this did not occur in wild-type worms upon P-80 supplementation. These results imply that knockdown of *ech-6* impairs amino acids catabolism which is exacerbated by fat supplementation.

### Fat diets affect the lifespan upon *ech-6* deficiency in a dose- and oleate-dependent fashion

**Figure 2:**
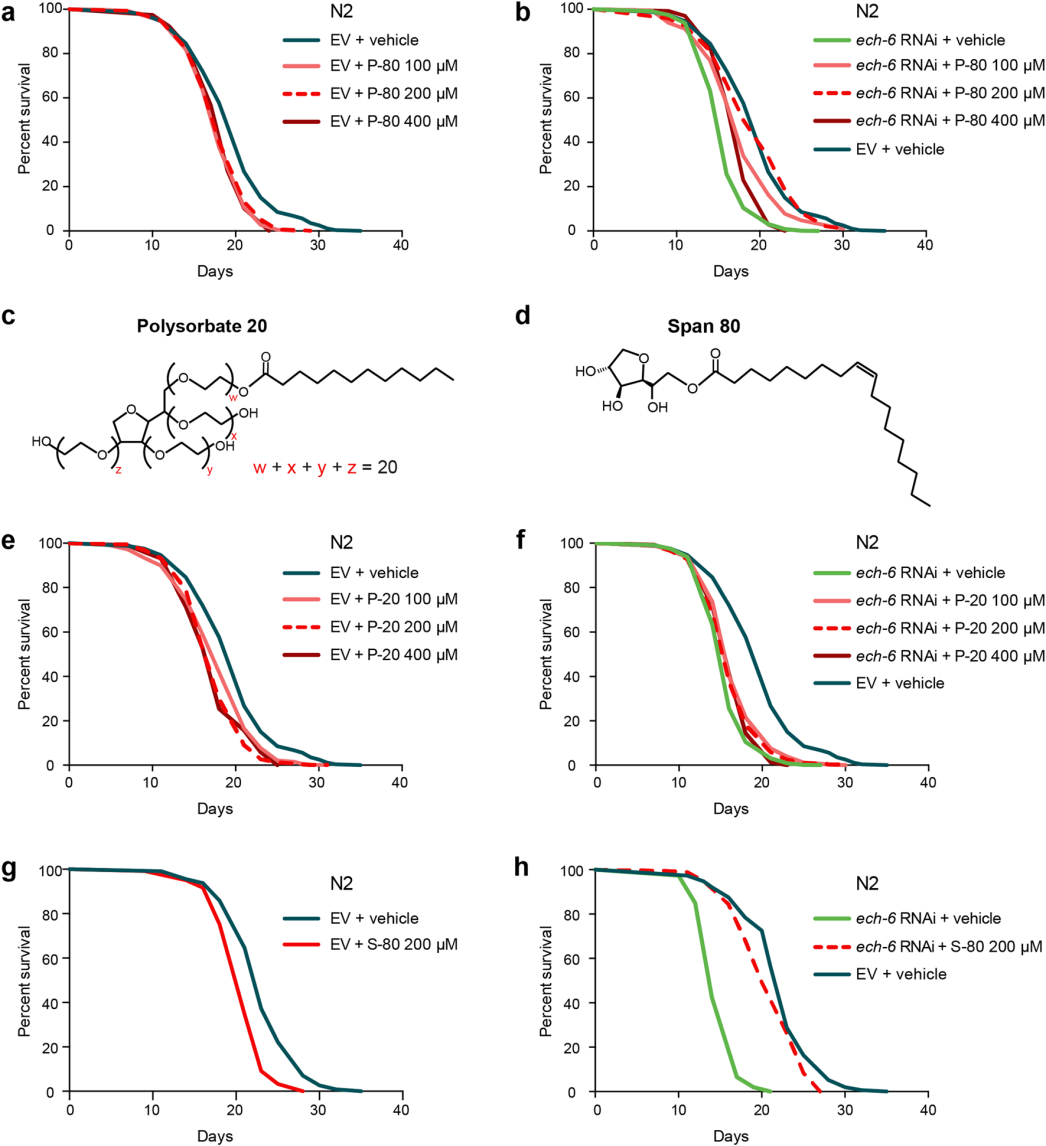
Fat diets affect the lifespan upon *ech-6* deficiency in a dose- and oleic acid-dependent fashion. (**a**) Lifespan analysis showing that P-80 supplementation at doses ranging from 100 µM to 400 µM reduces lifespan (*p* < 0.0001, log-rank test). (**b**) Lifespan analysis of worms upon *ech-6* deficiency showing that feeding *ech-6*-deficient worms the P-80 diet at 200 µM results in a maximum restoration of lifespan (*p* = 0.564*,* comparing *ech-6* RNAi + P-80 200 µM to empty vector (EV) + vehicle, log-rank test). (**c**) Chemical structure of Polysorbate 20 (P-20). P-20 consists of a hydrophilic group of ethylene oxide polymers and a lauric acid. (**d**) Chemical structure of Span 80 (S-80). S-80 consists of a sorbitan monoester and an oleic acid. (**e**) Lifespan analysis of worms fed with P-20 showing that worms exposed to the P-20 diet at doses ranging from 100 µM to 400 µM lived significantly shorter (*p* < 0.0001, log-rank test). (**f**) Lifespan analysis of worms upon *ech-6* deficiency showing that P-20 supplementation at a concentration from 100 to 400 µM does not prevent *ech-6* RNAi from shortening lifespan (*p* < 0.0001, log-rank test). (**g**) Lifespan analysis of worms fed a S-80 diet showing that S-80 supplementation at 200 µM shortens lifespan (*p* < 0.0001, log-rank test). (**h**) Lifespan analysis of worms upon *ech-6* deficiency showing that S-80 supplementation at 200 µM protects worms against *ech-6* RNAi-induced lifespan decrease (*p* < 0.0001, log-rank test). See also Table S1 for lifespan data.

Next, we determined the dose effect of P-80 on lifespan by supplementing worms with P-80 at increasing concentrations including 100 µM, 200 µM and 400 µM. Wild-type worms had a comparable decrease in lifespan when fed with three P-80 concentrations (Fig. 2a; Supplementary Table S1). In contrast, subjecting *ech-6*-deficient worms to a P-80 diet at the respective concentrations yielded distinct lifespan outcomes in which 200 µM P-80 resulted in the greatest lifespan normalization (Fig. 2b; Supplementary Table S1).

As the compound P-80 comprises a hydrophilic group of ethylene oxide polymers and an oleic acid moiety (Fig. 1a), we asked which constituents could be responsible for the lifespan phenotypes observed above. To test this, we made use of two other types of soluble fat, i.e. polysorbate 20 (P-20) and span 80 (S-80). P-20 consists of the same polar complex as P-80 but is bound to a saturated fatty acid with a shorter chain length, namely C12:0, lauric acid (Fig. 2c). S-80 is another oleic acid derivative containing a smaller polar moiety (Fig. 2d). Like the effects of P-80 on wild-type worms, supplementation of P-20 at 100 µM, 200 µM, and 400 µM shortened the lifespan of wild-type worms by a similar extent (Fig. 2e; Supplementary Table S1). In contrast to the effects of P-80 on worms with reduced *ech-6* expression, supplementation of P-20 at any of the three concentrations to *ech-6-*deficient worms did not reverse the shortened lifespan (Fig. 2f; Supplementary Table S1). Accordingly, we reasoned that rather than the hydrophilic polar complex of P-80, it is the oleic acid moiety that contributed to the restored lifespan of *ech-6*-deficient worms when fed with the dietary fat P-80. In line with this, supplementing *ech-6*-deficient worms with 200 µM S-80 extended their lifespan to near wild-type levels, while wild-type worms lived significantly shorter when fed with S-80 (Fig. 2g,h; Supplementary Table S1). Taken together, our results suggest that although different fat diets cause premature aging, *ech-6* specifically interacts with oleic acid-containing lipids to impinge on longevity.

### Knockdown of *ech-6* suppresses metabolic activity and energy-intensive processes including growth and mobility

**Figure 3:**
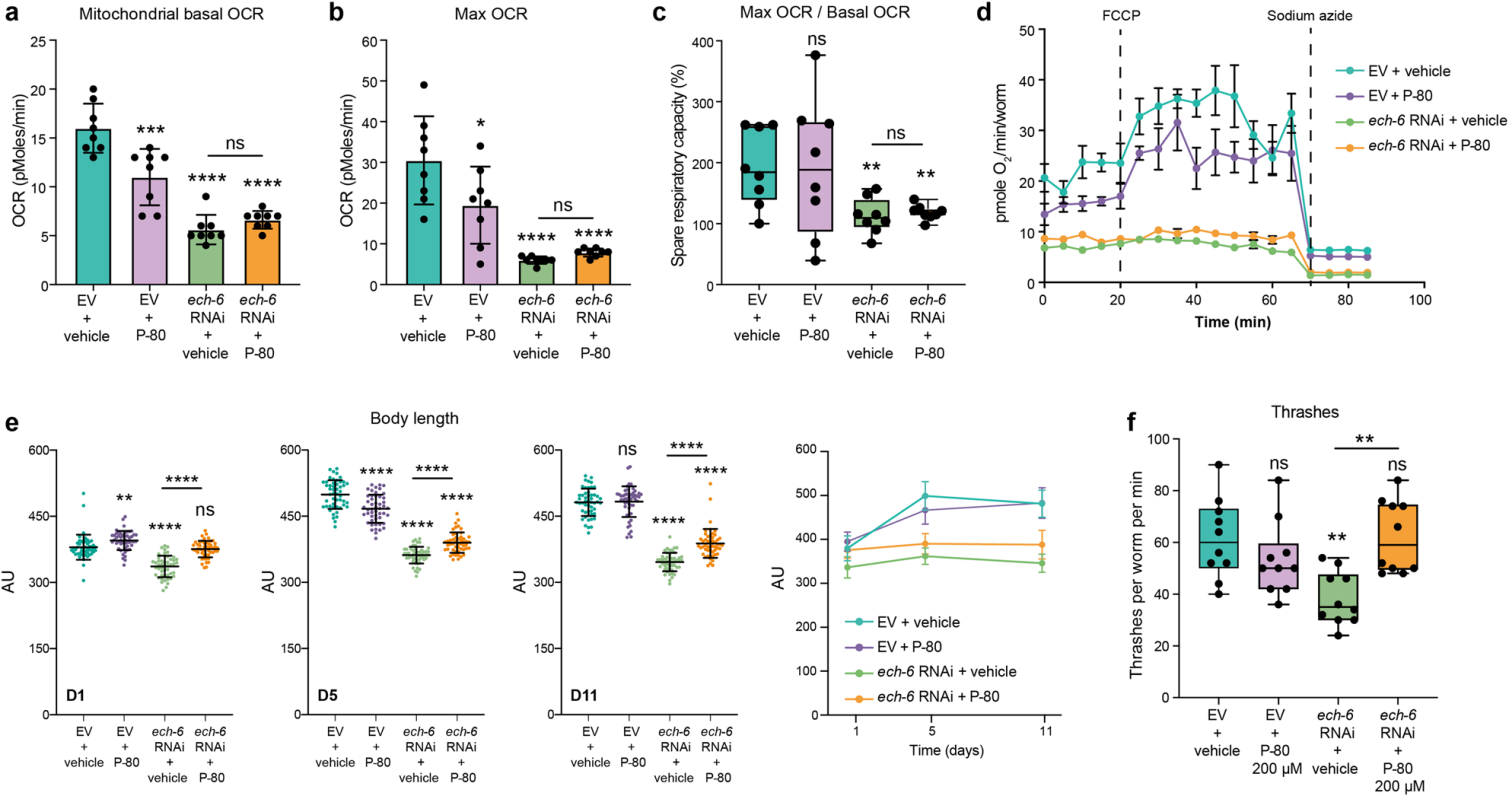
RNAi of *ech-6* suppresses metabolic activity, mobility, and growth. (**a**, **b**) Mitochondrial basal and maximum oxygen consumption rate (OCR) in adult N2 worms at day 5 of adulthood. P-80 supplementation and *ech-6* RNAi decrease mitochondrial respiration rate at both the basal (A) and the maximal levels (B). Mean ± SD of n = 8 biological replicates. (**c**) Mitochondrial spare respiratory capacity in percentage. (**d**) Raw averaged traces of oxygen consumption from day 5 adult N2 worms. Mean ± SEM (n = 8); FCCP, an uncoupler reagent, was added at the indicated time to achieve the maximum mitochondrial respiration, while sodium azide, a complex IV inhibitor, was added to fully block mitochondrial respiration, thereby measuring non-mitochondrial oxygen consumption. (**e**) Body length of N2 worms at day 1, day 5 and day11 of adulthood, respectively. Mean ± SD of n = 50 animals. P-80 supplementation ameliorates *ech-6* RNAi-mediated growth inhibition in three age groups of worms examined. (**f**) Thrashing of N2 worms at day 2 of adulthood exposed to *ech-6* RNAi and 200 µM P-80 diet, individually or in combination. P-80 supplementation to *ech-6*-deficient worms restores movement ability, measured by the frequency of thrashes per worm per minute. Mean ± SD of n = 10 animals. The bar graphs depict mean ± SD. An asterisk directly above a box refers to statistical significance compared to empty vector (EV)-treated wild-type fed a regular NGM diet. **p < 0.01; ***p < 0.001; ****p < 0.0001; ns, not significant; one-way ANOVA, Holm-Sidak correction.

Mitochondria provide the majority of cellular energy via oxidative phosphorylation which consumes oxygen and generates ATP^38^. To further characterize whether knockdown of *ech-6* or addition of fat affected mitochondrial capacity for energy production, we measured mitochondrial respiration by monitoring oxygen consumption rate (OCR) using Seahorse respirometry^39^. Supplementation of P-80 to wild-type worms reduced the level of basal and maximal mitochondrial respiration without altering spare respiratory capacity (Fig. 3a–d). In comparison, knockdown of *ech-6* reduced both mitochondrial respiration and spare respiratory capacity (Fig. 3a–d), indicating an overall deficiency in energy production. Feeding *ech-6*-deficient worms with P-80 did not show an additive effect (Fig. 3a–d).

Growth and movement are two physiological processes that heavily rely on cellular energy supply where mitochondria play a paramount role^38^. We therefore asked whether body length and motility were compromised due to *ech-6* deficiency or excessive fat intake. Indeed, wild-type worms subjected to the P-80 diet remained a bit smaller at day 5 of adulthood (Fig. 3e), whereas their mobility was not affected (Fig. 3f). In comparison, knockdown of *ech-6* reduced both the body size and the movement capacity (Fig. 3e,f), while supplementing P-80 to *ech-6*-deficient worms improved both parameters relative to that of *ech-6*-deficient worms (Fig. 3e,f). Taken together, our observations show that P-80 supplementation counteracts *ech-6* deficiency-caused suppression on growth and mobility, despite that it compromises mitochondrial respiration and growth in wild-type animals.

### Fat diet-induced alterations in lipid profiles are diminished in the context of *ech-6* knockdown

**Figure 4:**
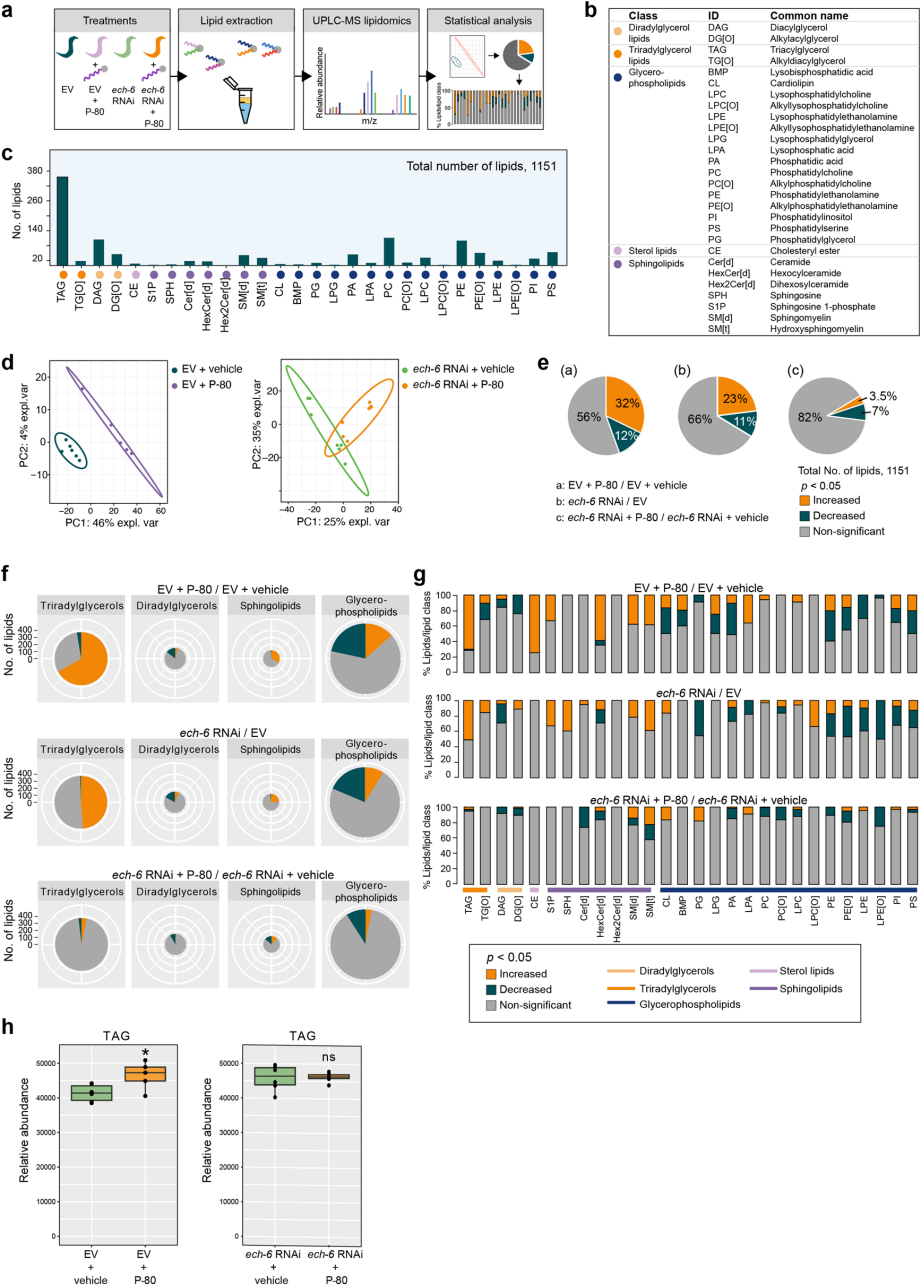
The effects of fat diets on lipid profiles are abrogated upon *ech-6* deficiency. (**a**) Experimental design. N2 worms were treated with *ech-6* RNAi or with P-80 from larval stage 1 and collected at day 1 adulthood for high-performance liquid chromatography-mass spectrometry (HPLC–MS)-based lipidomics. (**b**) Overview of worm lipids and distribution in the main lipid clusters. (**c**) The lipid composition of day-1 adult N2 worms. 28 lipid species and 1151 individual lipids were detected in worms. Triacylglycerides (TAG) make up the largest percentage (30.9%) of total lipids. (**d**) Partial least squares-discriminant analysis (PLS-DA) showing group separations based on significantly changed lipids by P-80 supplementation in empty vector (EV)- and *ech-6* RNAi-treated worms. (**e**) Pie charts depicting the percentages of increased and decreased lipids by P-80 supplementation or *ech-6* RNAi. A *p*-value < 0.05 was applied for determining statistical significance. (**f**) Statistical summary of significantly changed lipids in the lipid classes of triradylglycerols, diradylglycerols, sphingolipids, and glycerophospholipids. The total number of detected lipids in each lipid class is represented by the radius of pie plots, while the percentage of increased and decreased lipids in each category is indicated by the respective colours. The effects of P-80 supplementation on each lipid class are significantly abolished upon knockdown of *ech-6* by RNAi. A *p*-value < 0.05 was applied to determine statistical significance. (**g**) Percentage distribution of significantly changed lipids by P-80 supplementation across the lipid species in empty vector (EV)- and *ech-6* RNAi-treated worms. A *p*-value < 0.05 was applied to determine statistical significance. (**h**) The relative abundance of TAG upon P-80 supplementation. Supplementation of P-80 to empty vector (EV)-treated worms significantly increased TAG levels, which is absent in *ech-6*-deficient worms. *p < 0.05; ns, not significant; Student’s t test. See also Figs. S2 and S3 and Supplementary Tables S2 and S4.

Feeding wild-type worms with the fat diet P-80 shortened lifespan, while P-80 supplementation upon knockdown of *ech-6* exhibited inverse effects (Fig. 1). We hence asked whether the way in which dietary fat was metabolized in the two genetic backgrounds may be relevant to the differences observed in the life history traits. To do this, we profiled lipids for wild-type and *ech-6-*deficient worms exposed to the P-80 diet using UPLC-MS-based lipidomics (Fig. 4a). Across all worm samples, we detected 1151 distinct lipids belonging to 28 lipid species which can be further grouped into five main lipid classes consisting of diradylglycerol lipids, triradylglycerol lipids, glycerophospholipids, sterol lipids, and sphingolipids (Fig. 4b,c; Supplementary Table S2). Partial least squares discriminant analysis (PLS-DA) demonstrated that wild-type worms fed the P-80 diet were well separated from the control group fed a regular diet by the first two principle components (Fig. 4d). However, upon knockdown of *ech-6*, worms fed the P-80 diet overlapped with those fed a regular diet, suggesting that P-80 diet-induced lipid changes were diminished upon *ech-6* deficiency (Fig. 4d).

We further confirmed this finding by analyzing the percentage of lipids showing significantly altered levels after supplementation of P-80. When wild-type worms were fed with P-80, 369 lipids (32%) were increased while 141 (12%) were decreased (Fig. 4e; Supplementary Table S2). Although knockdown of *ech-6* also elicited changes in lipids relative to empty vector-treated wild-type controls (Fig. 4e; Supplementary Table S2), supplementation of P-80 to *ech-6*-deficient worms had a minor effect on lipid levels compared to *ech-6*-deficient worms fed a regular diet, with only 41 (3.5%) increased and 82 (7%) decreased (Fig. 4e; Supplementary Table S2).

To further determine the patterns of the change for each lipid class and lipid species, we analyzed the distribution of significantly changed lipids in triradylglycerols, diradylglycerols, sphingolipids and glycerophospholipids (Fig. 4f; Supplementary Table S2). The lipid classes triradylglycerols and glycerophospholipids had the greatest number of lipids affected by P-80 supplementation in wild-type worms (Fig. 4f; Supplementary Table S2). Next, we examined this distribution in detail in each lipid species (Fig. 4g; Supplementary Table S2). Percentage distribution of both increased and decreased lipids within each lipid species showed that triacylglycerols (TAG), cholesteryl ester (CE), and sphingolipids including sphinganine 1-phosphate (S1P), hexocylceramide (HexCer[d]), sphingomyelin (SM[d]), and hydroxysphingomyelin (SM[t]) comprised almost solely increased lipids when wild-type worms were fed with P-80 (Fig. 4g; Supplementary Table S2). Among those lipids, the total abundance of TAG was also significantly increased by P-80 supplementation in wild-type worms (Fig. 4h; Supplementary Table S2). In contrast, a large portion of glycerophospholipids was decreased under the same condition (Fig. 4f,g; Supplementary Table S2). These results imply that supplementation of P-80 to wild-type worms does not merely give rise to accumulation of storage lipids (i.e., TAG), but also profoundly change the profile of membrane lipids such as the glycerophospholipids.

Knockdown of *ech-6* altered lipid profiles in a similar fashion as did P-80 supplementation, i.e., triradylglycerols and sphingolipids comprised primarily increased lipids, while the majority of decreased lipids were enriched in glycerophospholipids (Fig. 4f,g; Supplementary Table S2). However, in contrast to the widespread effects of P-80 on wild-type worms, supplementation of P-80 had greatly reduced effects on nearly all lipid classes and species including TAG in *ech-6-*deficient worms (Fig. 4f–h; Supplementary Table S2). These results suggest that excessive fat intake in *ech-6*-deficient worms may be used as fuel for energy production, for instance through fatty acid β-oxidation, rather than being converted into triacylglycerols for storage or into glycerophospholipids for membrane synthesis.

To determine whether the effects of a fat diet on lipid profiles were specific to oleic acid-enriched fat, we measured lipid changes upon the addition of lauric acid-enriched fat P-20. Feeding wild-type worms with P-20 changed the pattern of overall lipid profiles in a way analogous to the effects of P-80, including a large number of increased triradylglycerols and decreased glycerophospholipids (Figs. S2a–c, S3; Supplementary Table S2 and S4). Supplementation of P-20 to *ech-6*-deficient worms affected a reduced number of triradylglycerols (Fig. S2b; Supplementary Table S2), however, other lipid classes including sphingolipids and glycerophospholipids were changed to a similar extent as seen in wild-type worms upon p-20 supplementation (Fig. S2b,c; Supplementary Table S2). Taken together, these results show that knockdown of *ech-6* renders animals resistant to overall lipid alterations caused only by oleic acid-rich fat diets like P-80.

### Energy production and lysosomal functions constitute the core set of upregulated biological processes upon fat supplementation

**Figure 5:**
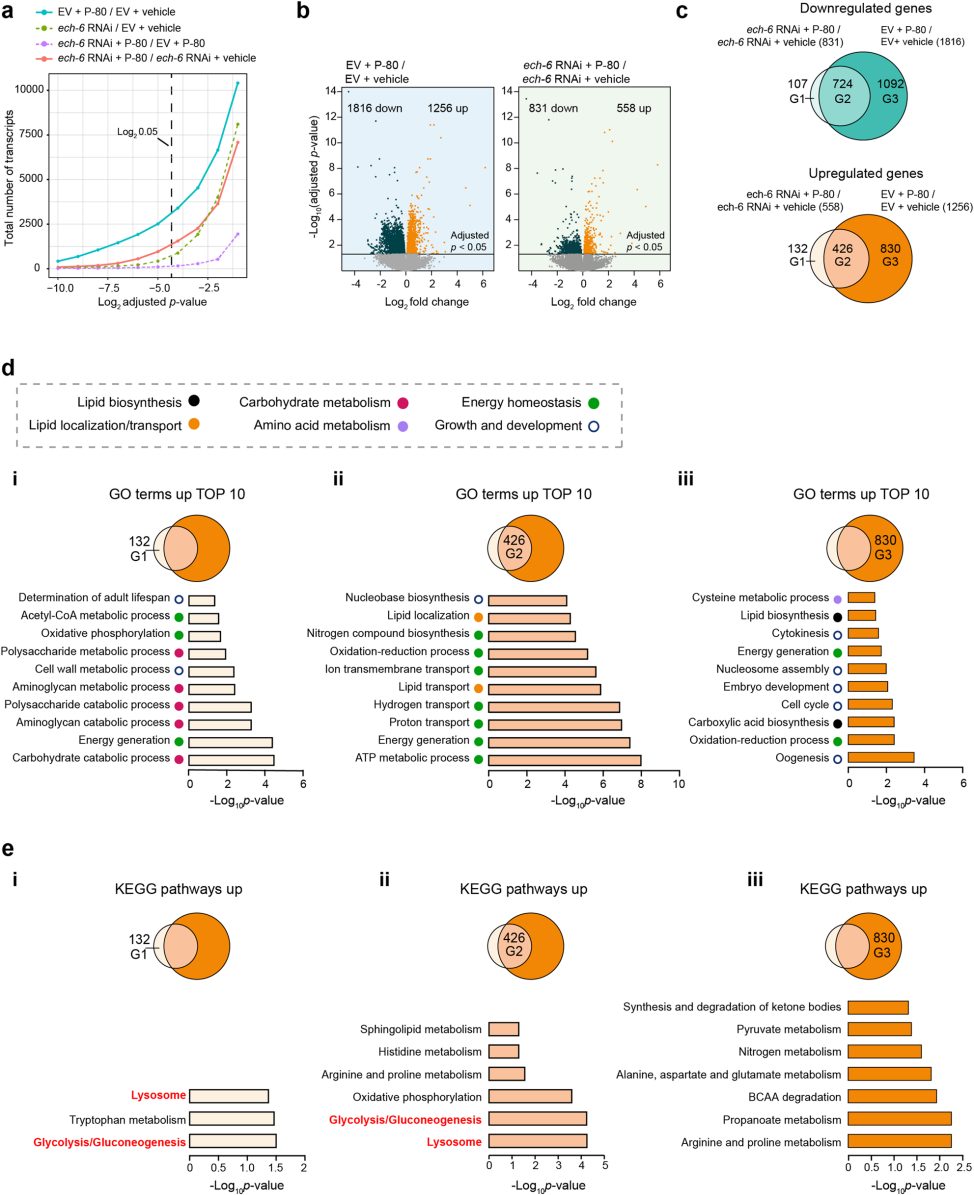
Dietary fat provokes activation of a core set of biological processes related to energy production and lysosomal functions. (**a**) The total number of transcripts change with adjusted *p*-values upon P-80 supplementation or depletion of *ech-6* by RNAi. An adjusted *p*-value was calculated for each transcript in the respective comparisons, i.e. between P-80-fed wild-type worms versus those fed a regular NGM diet, between *ech-6*-deficient versus wild-type worms, between P-80-fed *ech-6*-deficient versus P-80-fed wild-type worms, and between P-80-fed *ech-6*-deficient worms versus those fed a regular NGM diet. P-80 supplementation to empty vector (EV)-treated wild-type worms induces a great number of differentially expressed transcripts and the transcriptional effects of P-80 supplementation are reduced in the context of *ech-6* RNAi, as shown by the comparison between *ech-6* RNAi + P-80 and *ech-6* RNAi + vehicle. Adjusted *p*-values (Benjamini–Hochberg method) < 0.05 was used to determine the statistical significance of the difference. (**b**) Volcano plots depicting differentially expressed transcripts induced by P-80 supplementation in empty vector (EV)- and *ech-6* RNAi-treated worms. Adjusted *p*-values (Benjamini–Hochberg method) < 0.05 was applied to define significantly up- and downregulated genes. (**c**) Venn diagrams comparing overlaps between upregulated (downregulated) genes in empty vector (EV)- and in *ech-6* RNAi-treated worms. G1, differentially down- or upregulated genes exclusively in *ech-6*-deficient worms. G2, commonly down- or upregulated genes in empty vector (EV)- and *ech-6* RNAi-deficient worms. G3, down- or upregulated genes exclusively in empty vector (EV)-treated worms. (**d**) Gene ontology enrichment analysis of upregulated genes belonging to G1, G2, and G3, respectively. GO terms were considered significantly enriched for a modified Fisher’s Exact *p*-value < 0.05 (an EASE score). (**e**) KEGG pathway enrichment analysis of upregulated genes belonging to G1, G2, and G3, respectively. Pathways were considered significantly enriched for a modified Fisher’s Exact *p*-value < 0.05 (an EASE score). See also Fig. S5 and Supplementary Table S3.

To understand the underlying mechanisms accounting for the longevity effect resulting from the interaction between *ech-6* and fat diets, we conducted RNA sequencing (RNA-seq) analyses. Wild-type worms subjected to the P-80 diet had large numbers of transcripts differentially expressed (Fig. 5a; Supplementary Table S3), among which 1816 and 1256 transcripts were down- and upregulated (Fig. 5b; Supplementary Table S3). Upon knockdown of *ech-6*, the total number of transcripts affected by P-80 supplementation were decreased (Fig. 5a), with only 831 and 558 remaining down- and upregulated (Fig. 5b). In *C. elegans*, *ech-6* belongs to a family of enoyl-CoA hydratase genes that consist of 10 members (Fig. S4A). To rule out the cross-reactivity of *ech-6* RNAi with other enoyl-CoA hydratases, we examined the expression of all enoyl-CoA hydratase members upon knockdown of *ech-6* (Fig. S4A). We found that knocking down *ech-6* by RNAi effectively reduced the expression of *ech-6* alone but not any other enoyl-CoA hydratase (Fig. S4A), demonstrating the specificity of the RNAi-based depletion of *ech-6*. *ech-6* is organized in a transcriptional operon with three genes, including *gcc-2*, *T05G5.5* and *vps-53*. Upon *ech-6* RNAi, neither consistent nor significant reduction in these transcripts were detected, largely excluding the possibility that *ech-6* RNAi interferes with the transcriptional process of the neighboring genes (Fig. S4B).

Next, we asked to what extent the transcriptional changes induced by P-80 supplementation in wild-type worms were different from those upon knockdown of *ech-6*. We compared up- and downregulated transcripts by P-80 supplementation in wild-type worms to those in *ech-6*-silenced worms (Fig. 5c), which can be categorized into three groups: (G1) up- or downregulated transcripts exclusively in *ech-6*-silenced worms; (G2) up- or downregulated transcripts co-existing in both wild-type and *ech-6*-silenced worms; (G3) up- or downregulated transcripts specific to wild-type worms (Fig. 5c). Function enrichment analysis of the transcripts from each group revealed that catabolic pathways such as carbohydrate catabolic process, aminoglycan catabolic process and polysaccharide catabolic process were overrepresented exclusively in the upregulated genes in *ech-6*-silenced worms upon P-80 supplementation (G1) (Fig. 5d; Supplementary Table S3). Anabolic pathways including lipid biosynthesis and carboxylic acid biosynthesis were enriched among the upregulated genes exclusively present in wild-type worms (G3) (Fig. 5d; Supplementary Table S3). On the other hand, biological processes involved in energy homeostasis and lipid transport were enriched among the commonly upregulated transcripts in both wild-type and *ech-6*-silenced worms (G2) (Fig. 5d; Supplementary Table S3). Taken together, these results suggest that genes involved in energy homeostasis and lipid transport represent a core set of high-fat responsive genes, regardless of the expression of *ech-6*, while lipid biosynthesis and carbohydrate catabolism are conditionally upregulated, depending on *ech-6*.

To determine the specific pathways in which each group of upregulated genes was involved, we performed KEGG pathway enrichment analysis. Pathways involving lysosome, glycolysis/gluconeogenesis, and oxidative phosphorylation were identified as the top 3 most significantly enriched pathways among the commonly upregulated transcripts present in both wild-type and *ech-6*-deficient worms (G2) (Fig. 5e; Supplementary Table S3). Moreover, lysosome and glycolysis/gluconeogenesis were again overrepresented for the upregulated transcripts exclusively present in *ech-6*-deficient worms (G1) (Fig. 5e; Supplementary Table S3), highlighting the importance of these pathways upon *ech-6* deficiency upon fat supplementation. In addition to the two pathways, tryptophan metabolism was found as another upregulated pathway in the context of *ech-6* RNAi (Fig. 5e; Supplementary Table S3). As opposed to the specific upregulation of lysosome and glycolysis/gluconeogenesis upon knockdown of *ech-6* (Fig. 5e; Supplementary Table S3), a number of pathways regulating various aspects of metabolism were enriched among the upregulated transcripts specific to wild-type worms upon P-80 supplementation (G3) (Fig. 5e; Supplementary Table S3). Among those were pathways involving amino acid metabolism such as BCAA degradation, and alanine, aspartate and glutamate metabolism, short chain fatty acid propanoate metabolism, and pyruvate and ketone body metabolism (Fig. 5e; Supplementary Table S3). Functional enrichment analysis of the downregulated transcripts revealed few enrichments, except for processes related to growth and development, which were found in the downregulated gene clusters commonly present in both wild-type and *ech-6*-deficient worms (G2) (Fig. S5a) and in those exclusively in wild-type worms (G3) (Fig. S5b; Supplementary Table S3). Taken together, these results suggest that supplementation of P-80 rather upregulates energy production and lysosome-related processes upon *ech-6* deficiency, as opposed to a broader metabolic influence in wild-type worms involving stimulation of other processes such as lipid biosynthesis, various amino acid metabolic pathways, and pyruvate and ketone body metabolism.

### Upregulation of *lipl-4* underlies fat diet-mediated lifespan effects upon* ech-6* deficiency

**Figure 6:**
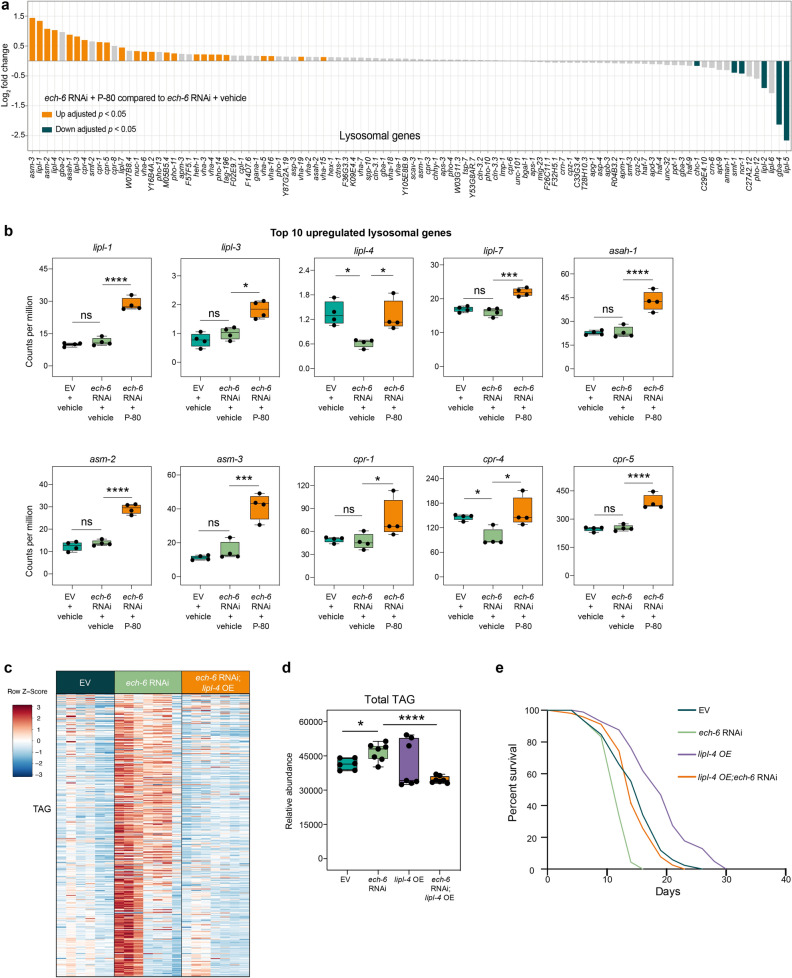
Overexpression of *lipl-4* promotes fat mobilization and restores the lifespan of *ech-6*-deficient worms. (**a**) Expressional changes of lysosome genes induced by P-80 supplementation in *ech-6*-deficient worms as compared to *ech-6*-deficient worms fed a regular NGM diet. 24 and 6 lysosome genes were respectively up- and downregulated. Adjusted *p*-values (Benjamini–Hochberg method) < 0.05 was considered significantly different. (**b**) Selection of the top 10 significantly upregulated transcripts in (A). 4 out of 10 upregulated transcripts encode lysosome lipases including *lipl-1*, *lipl-4*, *lipl-3*, and *lipl-7*. Adjusted *p*-values (Benjamini–Hochberg method) < 0.05 was considered significantly different. (**c**) Heatmap showing that overexpression of *lipl-4* decreases the level of triacylglycerols (TAG) upon knockdown of *ech-6* as compared to that in worms treated with *ech-6* RNAi alone. The lipid species of triacylglycerols were ordered from top to bottom by acyl chain length. (**d**) Quantification of the total amount of TAG showing that overexpression of *lipl-4* decreases the total TAG content upon knockdown of *ech-6* as compared to that in worms treated with *ech-6* RNAi alone. (**e**) Survival curves of worms overexpressing *lipl-4* subjected to *ech-6* RNAi showing that *lipl-4* overexpression restores the lifespan of *ech-6*-depleted worms to near-wild type levels (*p* = 0.2631, comparing *lipl-4* OE;*ech-6* RNAi to empty vector (EV), log-rank test). See also Table S1 for lifespan data and Table S2 for triacylglycerol measurement. *p < 0.05; **p < 0.01; ***p < 0.001; ****p < 0.0001; ns, not significant; one-way ANOVA.

Given that the lysosomal pathway emerged as one of the most significantly upregulated pathways by P-80 supplementation, particularly upon *ech-6* deficiency (Fig. 5e), we asked whether upregulation of lysosomal genes could be relevant to the effects of the P-80 diet on the lifespan of *ech-6*-deficient worms. To address this, we profiled the expression of lysosomal genes and found that the majority was significantly upregulated after P-80 supplementation to *ech-6*-deficient worms (Fig. 6a). To search for candidate genes that account for P-80-mediated longevity effect in *ech-6*-deficient worms, we focused on the top 10 upregulated lysosomal genes (Fig. 6b). 4 out of 10 genes encode lysosomal lipases including *lipl-1*, *lipl-3*, *lipl-4*, and *lipl-7*, while the other 6 genes are involved in sphingolipid metabolism and proteolysis, including *asah-1*, *asm-2*, and *asm-3* for sphingolipid metabolism, and *cpr-1, cpr-4,* and *cpr-5* for proteolysis (Fig. 6b). Lipases break down complex fat molecules such as triglycerides into their component fatty acids and glycerol molecules through hydrolysis of ester bonds so as to provide substrates for energy production or storage^40^. Given the fact that the ester bond linking oleate to the hydrophilic group in the P-80 compound is similar to that in triglycerides, we hypothesized that the upregulated lipases in *ech-6*-deficient worms after P-80 supplementation may facilitate the digestion of P-80 for an ultimate breakdown. The lysosomal lipase LIPL-4 is the most well-characterized in relation to fat metabolism and longevity in *C. elegans* and overexpression of *lipl-4* has been shown to promote fat mobilization and adjust mitochondrial activity^41–44^. Therefore, we asked whether overexpression of *lipl-4* mimics the effect of P-80 supplementation on the lifespan of *ech-6*-deficient worms by promoting endogenous fat mobilization. To do this, we developed an integrated version of a transgenic strain that expresses *lipl-4* under its own promoter^42,44^ and characterized their lipid profiles using UPLC-MS-based lipidomics. We found that overexpression of *lipl-4* in *ech-6*-deficient worms significantly reduced the level of almost all triglyceride species and that of total triglycerides content (Fig. 6c,d; Supplementary Table S2), indicating that these fats were released by overexpressing *lipl-4* for energy production. Given that feeding *ech-6*-deficient worms with P-80 restored the lifespan to a near-normal level, we next examined whether enhancing endogenous fat utilization through overexpression of *lipl-4* similarly benefits the lifespan of *ech-6*-deficient worms. Indeed, overexpressing *lipl-4* fully rescued the lifespan of *ech-6*-deficiency (Fig. 6e; Supplementary Table S1). Altogether, our data support that enhanced lysosomal lipolysis restores lipid mobilization and lifespan, similar to P-80 supplementation, when *ech-6* function is impaired.

## Discussion

In this study, we uncover the interactions between *ech-6* and fat diets in regulation of lifespan in *C. elegans*. Upon fat supplementation, decreasing the expression of *ech-6* rendered worms resistant to the deleterious effects of a fat-enriched diet on lifespan. However, under normal dietary conditions, knocking down *ech-6* shortened lifespan. The gene *ech-6* encodes a protein in BCAA catabolism. Knocking down *ech-6* resulted in amino acid accumulation and inhibited metabolic activity, thereby causing energy crisis and suppressing physiological activities that are dependent on energy homeostasis. Supplementation of fat to *ech-6*-deficient worms prevented the lifespan decrease and partially restored the physiological functions essential for growth and fitness. UPLC-MS-based lipidomics revealed that although dietary fat supplementation profoundly altered lipid profiles in wild-type worms particularly by increasing storage lipids triacylglycerols, the effect of fat-enriched diets on lipid content was abolished in *ech-6*-deficient worms. Using a transcriptomics approach followed by confirmation experiments, we identified that upregulation of lysosomal activity for instance by overexpression of lysosomal lipase *lipl-4* underlies the lifespan response to fat diets upon *ech-6* deficiency. Together, these results emphasize a role of ECH-6 in regulating metabolic flexibility by virtue of upregulation of lysosomal pathway in response to dietary fat overload and in turn affecting lifespan.

**Figure 7:**
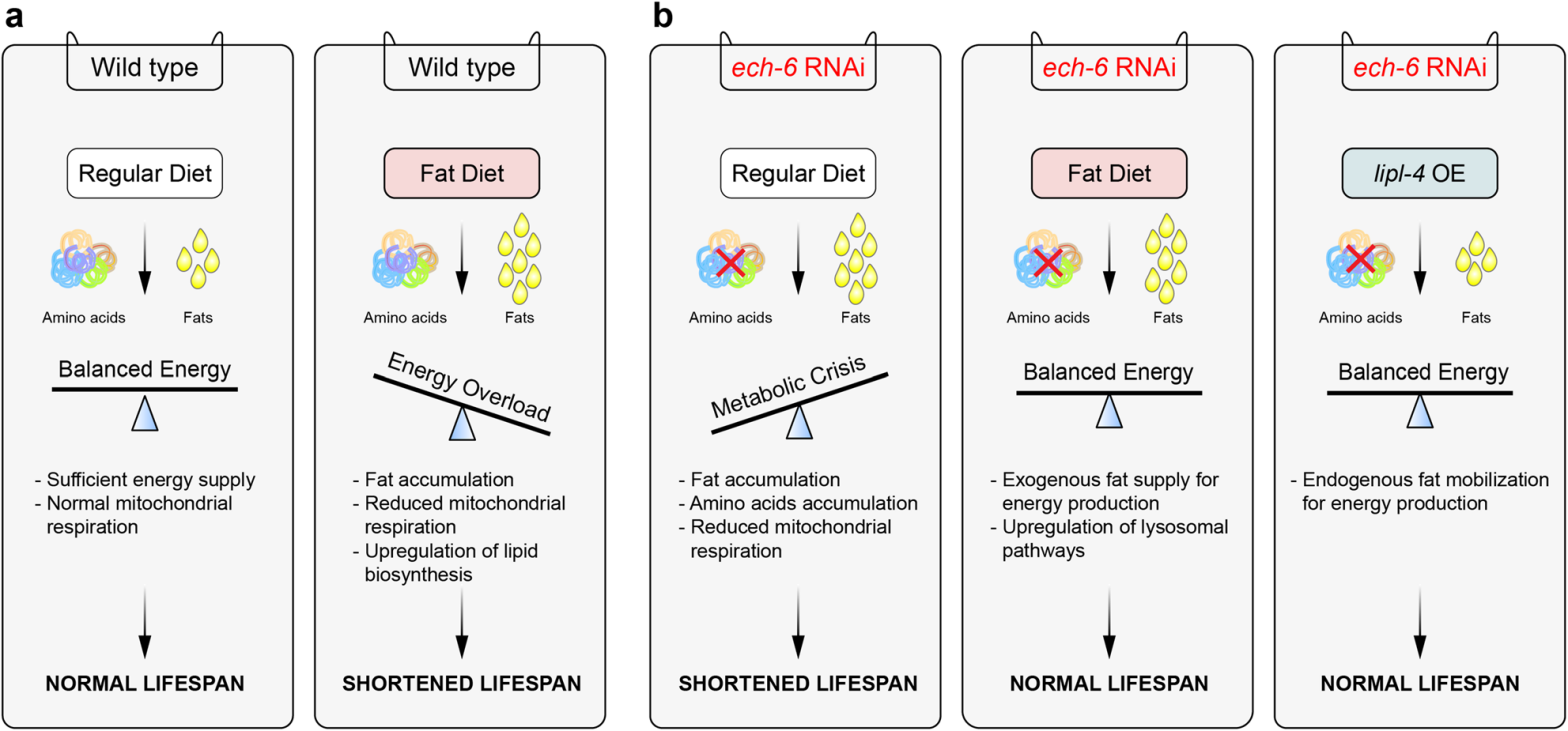
Model for hypothetic interactions between *ech-6, lipl-4,* a fat diet, and lifespan. (**a**) On a regular diet, worms have sufficient energy supply from mitochondria to maintain a homeostatic balance between energy production and energy consumption. As such, worms display a normal lifespan. However, when worms are exposed to excessive dietary fat, lipid overload disturbs energy homeostasis, suppresses mitochondrial respiration, causes fat accumulation, and ultimately leads to a shortened lifespan. (**b**) Knockdown of *ech-6* engenders metabolic crisis under normal dietary conditions by disrupting branched-chain amino acid degradation, inhibiting mitochondrial respiration, and resulting in fat and amino acids accumulation, which in turn causes insufficient energy production and lifespan reduction. This disrupted energy balance can be restored by either supplying dietary fat to *ech-6*-depleted worms or by enhancing endogenous fat mobilization for energy production through overexpression of *lipl-4*.

In this work, we unraveled a new connection between *ech-6* and fat-enriched diets in modulating aging through their interactive effects on energy metabolism in *C. elegans*. As illustrated in the model shown in Fig. 7, we suggest that worms grown on a normal diet are metabolically flexible, which enables them to switch efficiently between available nutrient sources and to maintain energy homeostasis^45^. This in turn leads to a normal lifespan (Fig. 7a). When worms are exposed to excessive dietary fat, this metabolic balance is interrupted, leading to reduced mitochondrial function, increased lipid biosynthesis and accumulated lipids, and in turn causing premature aging and shortened lifespan (Fig. 7a). In contrast, this situation is completely changed when the expression of *ech-6* is reduced in these worms. *ech-6* encodes an enoyl-CoA hydratase based on the homology with the human ECHS1 and is likely involved in the degradation of branched-chain amino acids valine and isoleucine^31,32^. We hence propose that upon knockdown of *ech-6—*when energy from branched-chain amino acids becomes unavailable—these worms go through an adaptive state that increases reliance on other nutrient sources such as fat (Fig. 7b). This hypothesis is supported by our findings that worms with reduced expression of *ech-6* do not accumulate fat when exposed to a fat-enriched diet (Fig. 4e–h). This adaptation allows them to cope better with the load of dietary fat and thereby to prevent premature aging induced by a fat-enriched diet (Fig. 7b). Knockdown of *ech-6* on the other hand leads to a metabolic crisis driven by disrupted BCAA degradation and suppressed mitochondrial respiration, in turn resulting in accelerated aging. However, this metabolic crisis is ameliorated by either supplying additional oleate-enriched fat to *ech-6*-deficient worms or by boosting endogenous fat mobilization for energy production through overexpression of *lipl-4* (Fig. 7b).

We found that knockdown of *ech-6* shortened lifespan which was prevented by supplementation of fat consisting of oleic acid rather than of lauric acid. We speculate that the different lifespan effects between the two fat diets could be due to two reasons: (1) P-20 and P-80 differ in their constituent fatty acids, oleic acid versus lauric acid and the latter of which releases around 1.5 times more ATP when fully broken down. Feeding *ech-6*-deficient worms with either fat diet had marginal effects on the level of TAG compared to the effects observed in wild-type worms upon dietary fat feeding. This suggests that the additional fat in *ech-6*-deficient worms was primarily catabolized through fatty acid β-oxidation. Therefore, due the lower capacity of P-20 to produce energy compared to that of P-80, it is possible that the concentration we tested in our study may not suffice to extend the lifespan of *ech-6*-deficent worms; (2) supplementing P-80 to *ech-6*-deficient worms had greatly reduced effects on glycerophospholipid profiles, whereas this attenuation was not observed when *ech-6*-deficient worms were fed with the P-20 diet. Glycerophospholipids are major components of membranes, alterations of which will inevitably affect various aspects of cell functions and lifespan.

We show that knockdown of *ech-6* substantially reduced lifespan^35^ and mitochondrial respiration. A closer investigation of this gene from a previous study showed the function of the encoded protein ECH-6 in degradation of propionate through the propionate shunt pathway^35^, in addition to the initially predicated function in the breakdown of BCAA in *C. elegans*^46^. Perturbations in the propionate shunt pathway, especially through depletion of *ech-6,* could result in accumulation of propionate and intermediates such as acrylyl-CoA—the substrate of ECH-6—to toxic levels^35^. Therefore, we presume that knockdown of *ech-6* incurs damages to mitochondria partially due to the production of those toxic metabolites.

Amino acids, particularly alanine, were significantly increased upon *ech-6* RNAi (Fig. S1). Alanine is one of 13 exclusively glucogenic amino acids that can feed into the TCA cycle. The increase in alanine upon *ech-6* RNAi may suggest a reduction in amino acids catabolism for energy metabolism. As to the significance of increase in alanine levels, previous studies have provided some insights. For example, increasing alanine levels through supplementation extends lifespan of *C. elegans*^47^. The metabolism from alanine to pyruvate may play a role as pyruvate supplementation also promotes longevity in *C. elegans*^48^. On the other hand, alanine supplementation in mice also showed protection against obesity due to high-fat diet feeding^49^. In line with these metabolomic data, the glutamate, alanine and branched chain amino acid catabolic pathways were upregulated in our RNAseq data upon P-80 supplementation (Fig. 5e). Although each of these amino acid pathways has been implicated in longevity^47,50,51^, in the context of P-80 supplementation we think this transcriptional upregulation is a result of cellular metabolic rewiring which warrants further study.

Our work shows that supplementation of P-80 and S-80, dietary fat that primarily contains oleic acid, shortened lifespan in *C. elegans*. In line with our findings, another study also reported that oleic acid supplementation negatively affected the lifespan of wild type N2 worms^52^. However, other studies that tested the lifespan effects of oleic acid have came to different conclusions, with both positive^36,53^ and no effects^54,55^ observed. Therefore, further studies are warranted to clarify whether the contradiction is the result of different concentrations of oleic acid used and/or different protocols of oleic acid supplementation adopted in these studies.

*Caenorhabditis elegans* are maintained on bacterial monocultures, commonly the *E. coli* OP50 and the *E. coli* HT115 in laboratory conditions. Bacterial metabolism of nutrients and drugs directly influences the treatment efficacy in the host^56–58^. In this study, we cannot exclude that the lifespan-shortening effects of P-80 supplementation are the result of a combined effect conferred by bacteria and *C. elegans* metabolism. Bacterial diets differ significantly in metabolite levels including fatty acids and amino acids^8^. Considering a lower level of oleic acid in *E. coli* HT115 compared to *E. coli* OP50^8^, it would be interesting to explore if supplementation of P-80 to *C. elegans* fed on a OP50 diet further enhances the lifespan shortening phenotype observed in *C. elegans* fed on a HT115 diet.

Knocking down genes by feeding worms with RNAi bacteria is the most convenient, efficient, and economic method, which is commonly used in *C. elegans*. However, this method can have limitations such as off-target and relative variability in the RNAi effects. In this study, to exclude the potential off-target effects, we showed that *ech-6* RNAi does not alter the transcripts of any other enoyl-CoA hydratase (Fig. S4A), nor of the neighboring genes (Fig. S4B). However, it remains important to validate our findings using *ech-6* mutant worms on the role of *ech-6* in lipid metabolism and lifespan.

Taken together, our work identifies *ech-6* as a potential regulator of metabolic flexibility modulating the susceptibility towards dietary fat overload. Although a substantial loss of *ech-6* expression or function is deleterious to lifespan in both *C. elegans* and humans, it is appealing to speculate that moderate suppression of this gene such as through pharmacological approaches could improve metabolic flexibility towards fat diet without compromising health and survival. We believe that this project opens up new avenues to elucidate longevity genes involved in GxE interactions.

## Materials and methods

### *Caenorhabditis elegans* strains and bacterial feeding strains

The *C. elegans* N2 (Bristol) and *E. coli* OP50 was obtained from the *Caenorhabiditis* Genetics Center (CGC). The LIPL-4 OE strain (LRL21) is an integrated version of the GR1971 strain (mgEx779[lipl-4p/K04A8.5p::lipl-4/K04A8.5::SL2::gfp + myo-2p::mcherry])^42^ that was backcrossed 4 times to wild-type. RNAi bacterial clones are *E. coli* HT115 strains, including the clone to knock down *ech-6 (*T05G5.6*)* which was derived from Ahringer library^59^. The *ech-6* RNAi clone was confirmed by sequencing. In this study, RNAi experiments initiated at larval stage 1.

### Nematode growing conditions and RNAi experiments

*Caenorhabditis elegans* were cultured and maintained on nematode growth media (NGM) at 20 °C. Worms of each strain were cultured on plates seeded with OP50 strain *Escherichia coli*. In RNAi experiments, synchronized N2 at L1 stage were obtained by alkaline hypochlorite treatment of gravid adults and transferred onto NGM plates (containing 2 mM IPTG and 25 mg/mL carbenicillin) seeded with *E. coli* HT115 containing empty vector or *ech-6* RNAi bacteria.

### Supplementation of polysorbate 80 (P-80), span 80 (S-80) or polysorbate 20 (P-20)

Pure P-80 (Merck KGaA, catalogue number: 8.22187.0500, lot number: S736868771), S-80 (Croda) or P-20 (Merck KGaA, Catalogue number: 8.22184.0500, Lot number: S7514484809) was added into the liquid NGM agar medium before autoclaving to obtain a final concentration of 100 µM, 200 µM, or 400 µM.

### Lifespan measurements

Worm lifespan was performed as described previously^18^. In short, a synchronized population of L1 worms of each strain was obtained as described above and seeded onto NGMi (containing 2 mM IPTG and 25 mg/mL carbenicillin) plates or NGMi plates containing different concentrations of P-80, S-80 or P-20. After worms reached the last larval stage L4, these worms were then transferred onto NGMi (or NGMi + P-80/S-80) plates containing 10 µM 5-FU. Worms were scored every other day. Worms that did not react to gentle stimulation were scored as dead. Worms that crawled off the plates or displayed a protruding vulva phenotype were censored.

### Analysis of fatty acid profile using targeted mass spectrometry-based platform

Fatty acid extraction was performed as mentioned in our previous study^8^. A synchronized population of 2000 L1 worms were cultured on plates seeded with *E. coli* HT115 or *ech-6* RNAi bacteria for 2.5 days until reaching young adult stage. Worms were then collected by washing off the culture plates with M9 buffer followed by three times of washing with dH_2_O. Worm pellet was transferred to a 2 mL Eppendorf tube using a glass pipette followed by snap-freezing in liquid N_2_ and freeze-drying overnight. Worm lysate was generated using a TissueLyser II (Qiagen) (adding a 5 mm steel bead and ice-cold 0.9% NaCl solution to a dried worm pellet) for 2 × 2.5 min at a frequency of 30 times/sec. The lysate was homogenized further by a tip sonication (energy level: 40 J; output: 8 watts) on ice water. BCA assay was used for protein quantification and used for sample normalization. A 150 µg worm protein lysate was transferred in a 4 mL fatty acid free glass vial followed by adding 1 mL of freshly prepared mixture of pure acetonitrile/37% HCl (ratio 4:1, v/v) to the lysate. Deuterium-labeled internal standard mixture (5.04 nmol d5-C18:0, 2.52 nmol d4-C24:0, and 0.25 nmol d4-C26:0) was added to each vial and then the fatty acid samples were hydrolyzed by incubating at 90 °C for 2 h. After the samples were cooled down to room temperature, 2 mL hexane was added to each vial and sample were mixed by vortexing for 5 s. After 1 min centrifugation at 1000 g, the upper layer was transferred to a fatty acid free glass tube and evaporated at 30 °C under a flow of nitrogen. Fatty acid pellets were dissolved in 150 µL final solution (chloroform–methanol-water, 50:45:5, v/v/v) containing 0.0025% aqueous ammonia and then transferred to a Gilson vial for ESI–MS analysis.

### Amino acid extraction and UPLC-MS/MS analysis

Amino acid extraction and UPLC-MS/MS were performed as previously described in our study^8^. In brief, 1 mL of 80% acetonitrile plus 20 µL of internal standard mixture containing 68 nmol d4-alanine, 44 nmol d3-glutamate, 40 nmol d3-leucine, 28 nmol d5-phenylalanine, 34 nmol d8-valine, 34 nmol d3-methionine, 26 nmol d4-tyrosine, 22 nmol d5-tryptophan, 46 nmol d3-serine, 48 nmol d7-proline, 24 nmol d7-arginine, 28 nmol d5-glutamine, 32 nmol d4-lysine, 26 nmol ^13^C-citrulline, 28 nmol d6-ornithine, 42 nmol d10-isoleucine, and 46 nmol d3-aspartate) were added to worm lysate and homogenized by vortexing in a 2 mL Eppendorf tube. Samples were centrifuged for 10 min at 4 °C at 16,000×*g* and the supernatant was transferred to a 4 mL glass vial and evaporated under nitrogen stream at 40 °C. Subsequently, the residue was dissolved in 220 µL of 0.01% (v/v in MQ water) heptafluorobutyric acid for UPLC-MS/MS analysis.

### Analysis of worm body length

After exposure to the various conditions, worms were washed with M9, and imaged with a Leica M295 microscope. Measurements were performed with Image J freeware (W.S. Rasband, U.S.A. National Institutes of Health, Bethesda, Maryland, USA, http://rsb.info.nih.gov/ij/, 1997–2012). Measurements were performed on 50 worms for each condition.

### Thrashing assay

After being treated with *ech-6* RNAi bacteria and 200 µM P-80, 10 adult worms at day 2 were used for the thrashing assay of each condition. In brief, a single worm was placed in a drop of M9 buffer on a clean glass slide and allowed to acclimatize for 30 s. The frequency of body bends was counted for 30 s as described previously^60^.

### Oxygen consumption rate analysis

Worm oxygen consumption rate (OCR) was measured using the Seahorse XF96 (Seahorse Bioscience) as previously described^39^. In brief, worms were cultured on plates with different conditions and washed off with M9 buffer after reaching day 1 adulthood. After three times of additional washing with M9 buffer, worms were transferred in 96-well Seahorse plates and OCR was measured six times. FCCP and sodium azide treatments were performed at a final concentration of 10 µM and 40 mM, respectively. Both mitochondrial (basal) and maximum OCR was measured for each condition.

### One-phase lipidomic extraction and lipidomics in *C. elegans*

Worms were synchronized at L1 and subjected to *ech-6* RNAi bacteria, 200 µM P-20, or 200 µM P-80 for 2.5 days until reaching day-1 adult stage. Lipidomics was performed as described^8,61^. Briefly, samples containing approximately 2000 worms were lyophilized in 2 mL tubes. The samples were homogenized and lipids extracted in 1:1 (v/v) methanol:chloroform using water bath sonication for 10 min in presence of internal standards, these were: Bis(monoacylglycero)phosphate BMP(14:0)_2_ (0.2 nmol), Cardiolipin CL(14:0)_4_ (0.1 nmol), Cholesterol ester CE(16:0)-D7 (2.5 nmol), Diacylglyceride DG(14:0)_2_ (0.5 nmol), Lysophosphatidicacid LPA (14:0) (0.1 nmol), Lysophosphatidylcholine LPC(14:0) (0.5 nmol), Lysophosphatidylethanolamine LPE(14:0) (0.1 nmol), Lysophosphatidylglycerol LPG (14:0) (0.02 nmol), Phosphatidic acid PA(14:0)_2_ (0.5 nmol), Phosphatidylcholine PC (14:0)_2_ (2 nmol), Phosphatidylethanolamine PE (14:0)_2_ (0.5 nmol), Phosphatidylglycerol PG (14:0)_2_ (0.1 nmol), Phosphatidylinositol PI (8:0)_2_ (0.2 nmol), Phosphatidylserine PS (14:0)_2_ (5 nmol), Ceramide phosphocholines SM (d18:1/12:0) (2.125 nmol), Triacylglyceride TG(14:0)_3_ (0.5 nmol) (Avanti Polar Lipids, Alabaster, AL). After centrifugation at 16,000×*g* at 4 °C for 10 min, the supernatant was collected in glass vials and dried under a stream of nitrogen gas at 45 °C and reconstituted in 150 µL 1:9 (v/v) methanol:chloroform. Chromatography was performed on a Dionex Ultimate 3000 binary UHPLC (Thermo Scientific) and on normal- and reversed phase polarity. MS data were acquired using negative and positive ionization using continuous scanning over the range of m/z 150 to m/z 2000. Data were analyzed using an in-house developed metabolomics pipeline written in the R programming language (http://ww.r-project.org). In brief, it consisted of the following five steps: (1) pre-processing using the R package XCMS, (2) identification of metabolites, (3) isotope correction, (4) normalization and scaling and (5) statistical analysis. All reported lipids were normalized to corresponding internal standards according to lipid class, as well as total protein content in samples, determined using a PierceTM BCA Protein Assay Kit. Lipid identification has been based on a combination of accurate mass, (relative) retention times, and the injection of relevant standards.

### Isolation of mRNA

For isolation of total mRNA, approximately 500 day^−1^ adult worms were collected in quadruplicates for each treatment. In brief, worm pellets were homogenized in TRIzol (Invitrogen) with a 5 mm steel metal bead and the isolation was continued according to manufacturer’s protocol. For RNAseq, genomic DNA residues were removed using RNase-Free DNase (QIAGEN) and samples were cleaned up with the RNeasy MinElute Cleanup Kit (QIAGEN).

### Library preparation

RNA libraries were prepared and sequenced with the illumina platform by Genome Scan (Leiden, Netherlands). Samples were processed for Illumina using the NEBNext Ultra Directional RNA Library Prep Kit (NEB #E7420) according to manufacturer’s description. Briefly, rRNA was depleted using the rRNA depletion kit (NEB# E6310). Subsequently, a cDNA synthesis was performed in order to ligate with the sequencing adapters. Quality and yields after sample preparation were measured with the Fragment Analyzer (Agilent). Sizes of the resulting products was consistent with the expected size distribution (a broad peak between 300–500 bp). Clustering and DNA sequencing using the Illumina cBot and HiSeq 4000 was performed according to manufacturer’s protocol with a concentration of 3.0 nM of DNA. HiSeq control software HCS v3.4.0, image analysis, base calling, and quality check was performed with the Illumina data analysis pipeline RTA v2.7.7 and Bcl2fastq v2.17.

### Read mapping, statistical analyses, and data visualization

Reads were subjected to quality control FastQC^62^ trimmed using Trimmomatic v0.32^63^ and aligned to the *C. elegans* genome obtained from Ensembl, wbcel235.v91 using HISAT2 v2.0.4^64^. Counts were obtained using HTSeq (v0.6.1, default parameters)^65^ using the corresponding GTF taking into account the directions of the reads. Statistical analyses were performed using the edgeR^66^ and limma/voom^67^ R packages. All genes with more than 2 counts in at least 4 of the samples were kept. Count data were transformed to log2-counts per million (logCPM), normalized by applying the trimmed mean of M-values method^66^ and precision weighted using voom^68^. Differential expression was assessed using an empirical Bayes moderated t test within limma’s linear model framework including the precision weights estimated by voom^67,68^. Resulting *p*-values were corrected for multiple testing using the Benjamini–Hochberg false discovery rate. Genes were re-annotated using biomaRt using the Ensembl genome databases (v91). Data processing was performed using R v3.4.3 and Bioconductor v3.5. The RNA-seq data are available at the Gene Expression Omnibus (GEO) Database under the ID GSE157701 and token: kvqlgiccbpczbct.

### Functional annotation of gene sets

Gene sets were analyzed for functional enrichments using the DAVID bioinformatics resource version 6.7^69^. Functional annotation clustering was performed using DAVID defined default settings incorporating gene sets from Gene Ontologies (biological process, cellular component, and molecular function), functional categories (including Clusters of Orthologous Groups (COG) ontologies, SP keywords, UP seq features), pathways (including KEGG), and protein domains (including INTERPRO PIR superfamily and SMART). The measured transcriptome was used as the background dataset for the enrichment tests and ReviGo^70^ was used to eliminate redundant GO terms for purposes of visualization and summarization. Venn diagrams were generated using BioVenn^71^.

### Quantification and statistical analysis

Statistical details, the number of biological replicates and any other forms of quantification present are specified in the respective figure legends and results section. Statistical analyses for lifespan, fatty acids, amino acids, mitochondrial respiration, body length and mobility were performed using the Prism 7 software (GraphPad Software, La Jolla, CA, USA). All comparisons of means were accomplished using a one-way ANOVA and two sample unpaired t-test as indicated in the corresponding figure legends. The significance *p*-values were adjusted to correct for multiple testing using the Holm-Sidak method. All other statistics were specified in each respective methods section unless otherwise noted. Gene expression was considered differential relative to respective control groups using adjusted *p*-values (Benjamini–Hochberg method) lower than 0.05. Lipid level was considered altered relative to respective control groups using *p-*values lower than 0.05. For lifespan studies, survival curves were calculated using the log-rank (Mantel-Cox) method.

## Supplementary Information

Supplementary Figures.

Supplementary Table S1.

Supplementary Table S2.

Supplementary Table S3.

Supplementary Table S4.

Supplementary Table S5.

## Data Availability

The accession number for the *C. elegans* RNA sequencing data reported in this paper is GEO: GSE157701 and GEO reviewer token is kvqlgiccbpczbct. The processed and normalized RNAseq data is also available as Table S3.
