# Supplementary material for: Reduced ech-6 expression attenuates fat-induced lifespan shortening in C. elegans
Source: Sci Rep. 2022 Mar 1;12:3350. doi: 10.1038/s41598-022-07397-9 (PMC8888598; doi:10.1038/s41598-022-07397-9)
Supplement: Supplementary file 1 — Supplementary Figures. [file 41598_2022_7397_MOESM1_ESM.docx]

**Supplemental figures and legends**

**
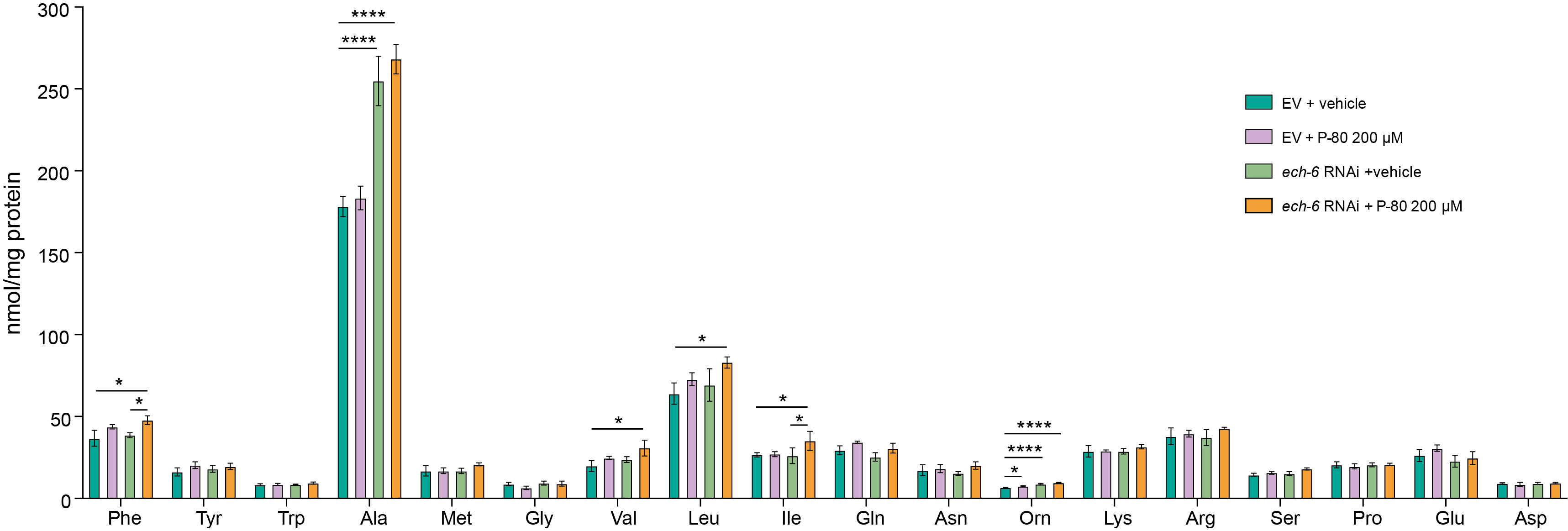
**

**Figure S1.** Amino Acid Profiles upon *ech-6* Depletion and Fat Supplementation, Individually or in Combination

Knockdown of *ech-6* significantly increases the levels of valine and ornithine, while supplementation of P-80 to *ech-6*-silenced worms increases the levels of branched-chain amino acids (valine, leucine, and isoleucine), phenylalanine, and ornithine as compared to the empty vector (EV) controls. **p* < 0.05; ***p* < 0.01; ****p* < 0.001; *****p* < 0.0001; one-way ANOVA; Holm-Sidak correction.

**
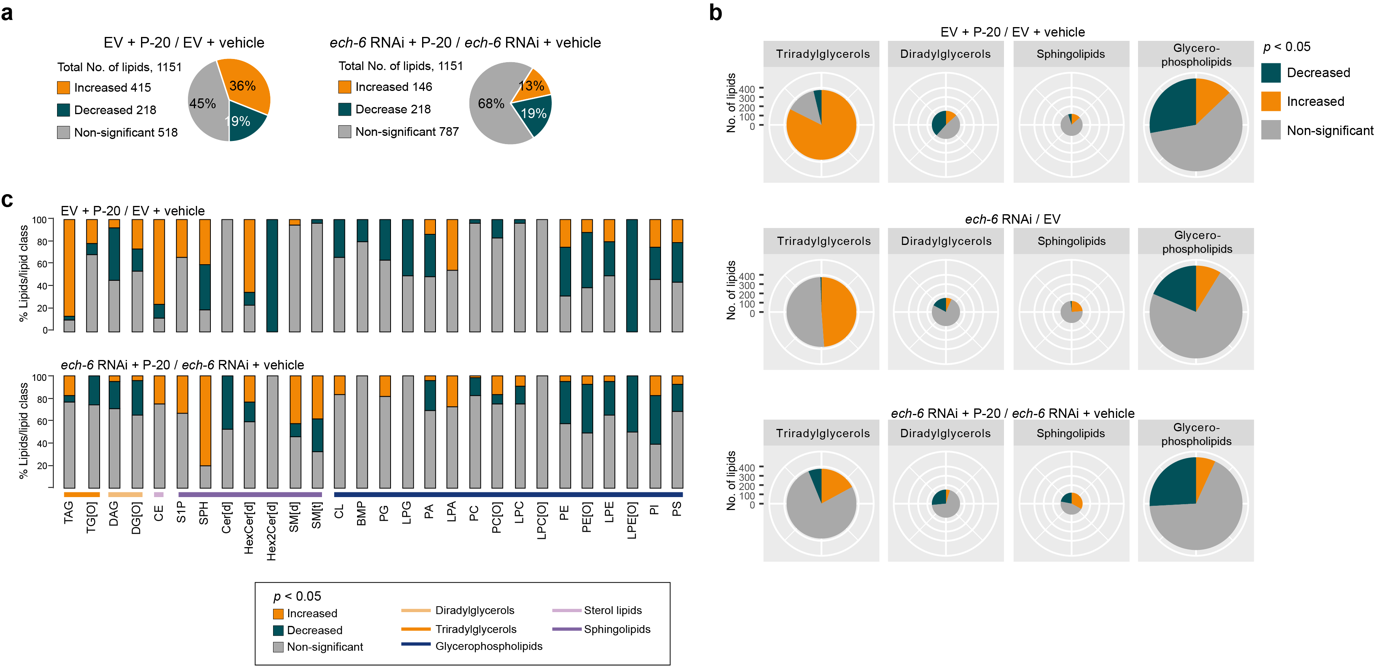
**

**Figure S2.** Knockdown of *ech-6* Diminishes the Effects of P-20 Supplementation on TAG but Not on Glycerophospholipid Profiles

(a) Pie charts depicting the percentages of increased and decreased lipids by P-20 supplementation. A *p*-value < 0.05 was applied for determining statistical significance.

(b) Statistical summary of significant changed lipids in the lipid classes of triradylglycerols, diradylglycerols, sphingolipids, and glycerophospholipids. The number of detected lipids in each lipid class is represented by the radius of pie plots, while the percentage of increased and decreased lipids in each category is indicated by the respective colours. Supplementation of P-20 shows reduced effects on triradylglycerols in *ech-6*-deficient worms compared to its effects in empty vector (EV)-treated wild-type worms, whereas other lipid classes are altered to a similar extent in empty vector (EV)- and *ech-6* RNAi-deficent worms. A *p*-value < 0.05 was applied to determine statistical significance.

(b) Percentage distribution of significantly changed lipids by P-20 supplementation across the lipid species in empty vector (EV)- and *ech-6* RNAi-deficient worms.


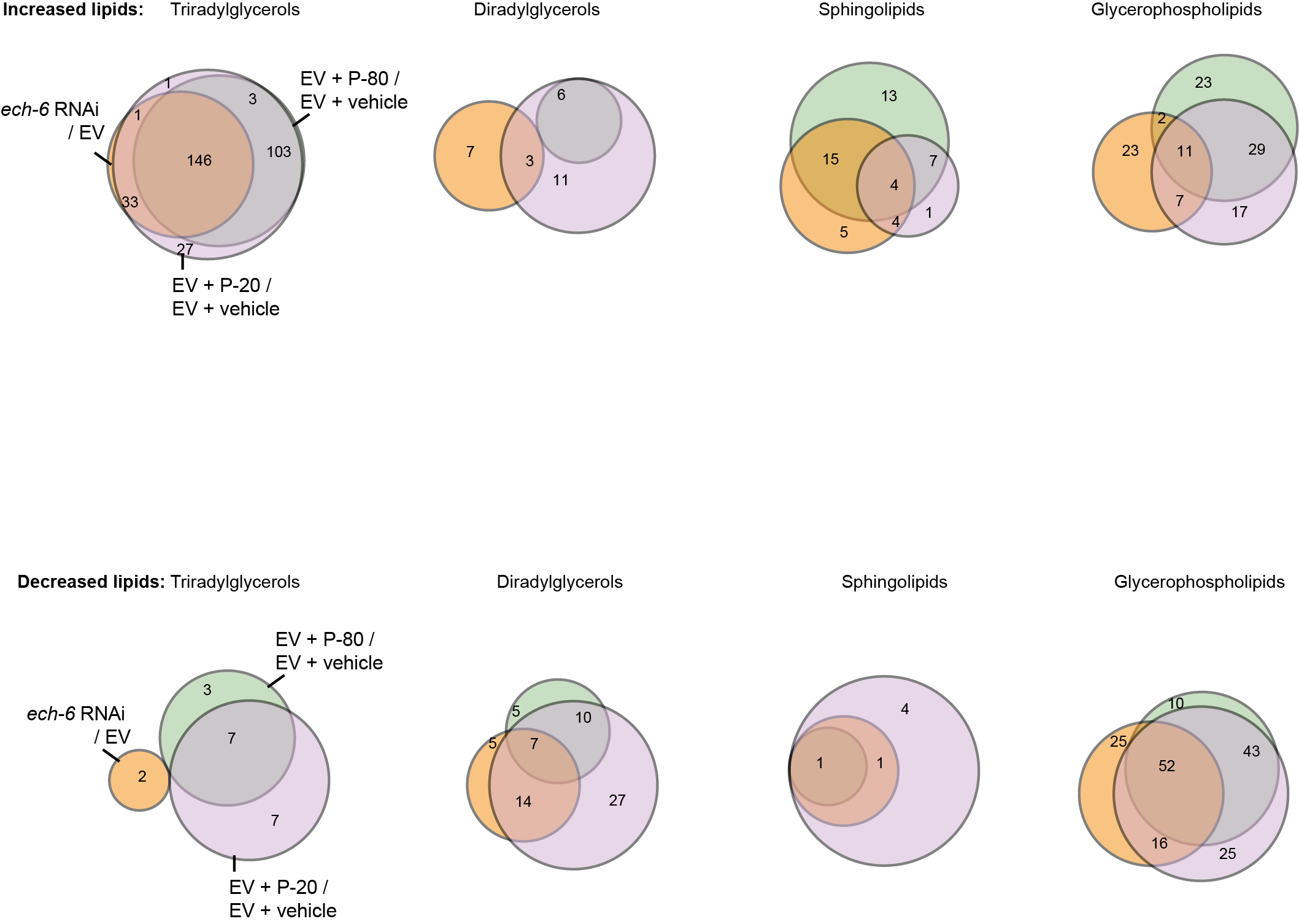


**Figure S3.** Venn diagram depicting overlap of significantly changed lipids in groups treated with RNAi against *ech-6* or supplemented with either P-80 or P-20. A *p*-value < 0.05 was applied for determining statistical significance.


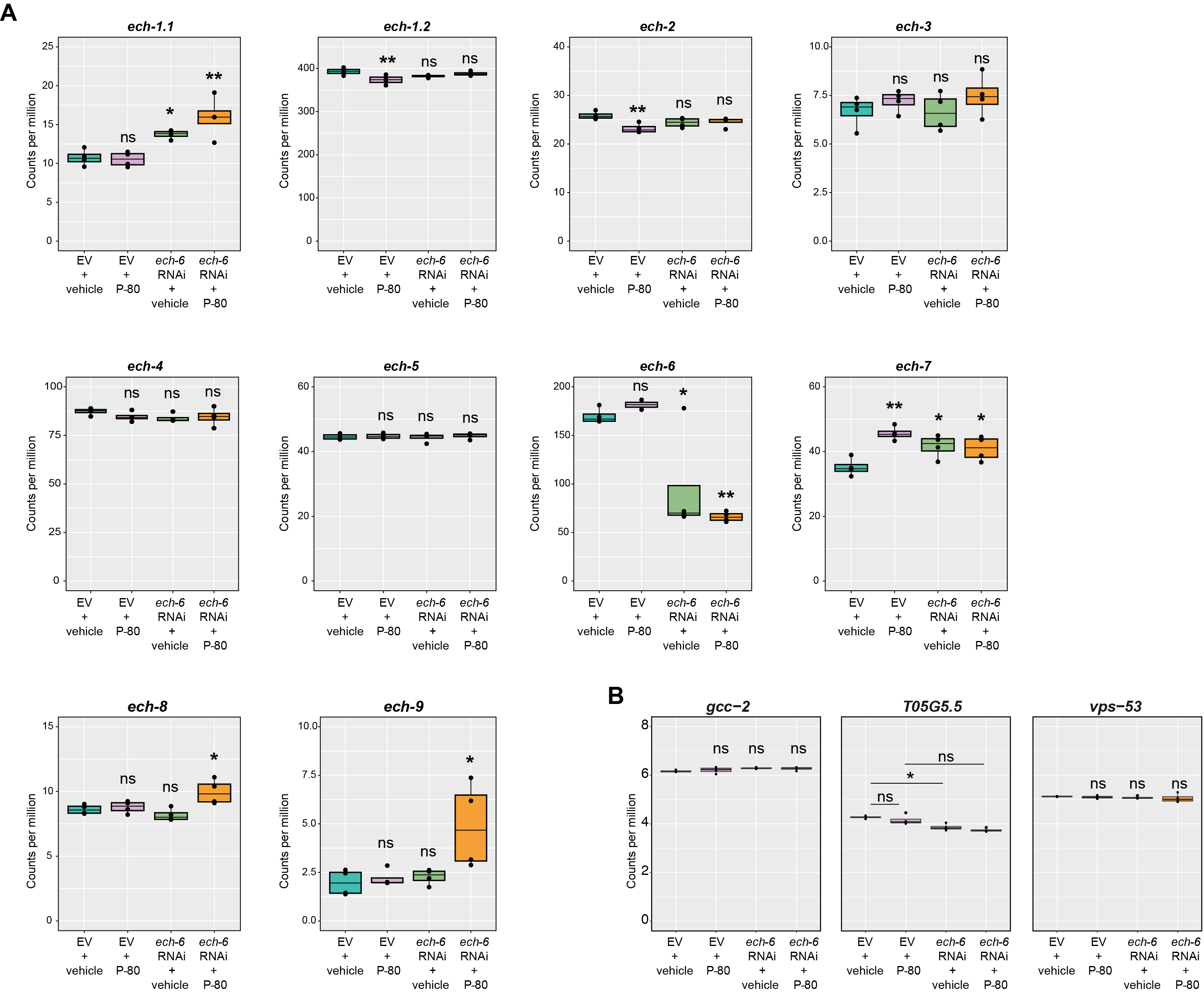


**Figure S4.** Expression of enoyl-CoA hydratase genes and genes residing in the same operon as *ech-6* upon *ech-6* knockdown. Knocking down *ech-6* by RNAi specifically reduced the expression of *ech-6* but not any other enoyl-CoA hydratases. **p* < 0.05; ***p* < 0.01; ns, not significant; one-way ANOVA.

**
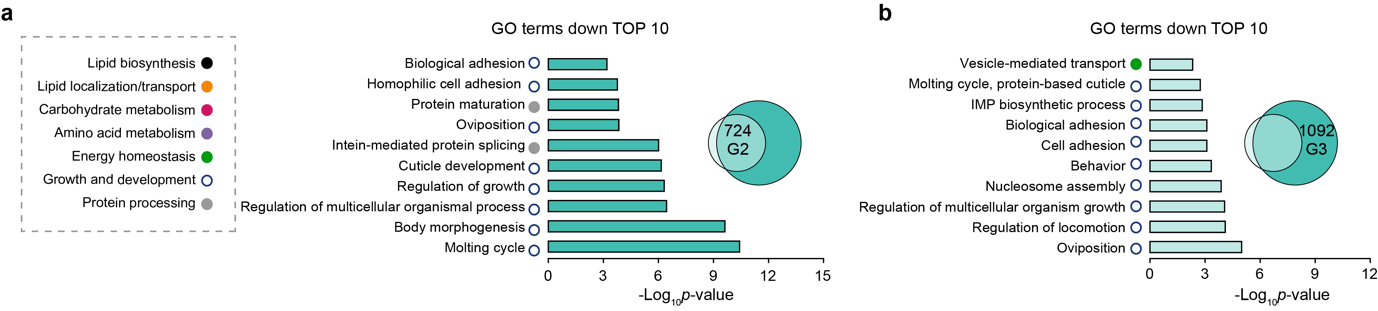
**

**Figure S5.** Gene Ontology Enrichment Analysis of Downregulated Genes by Fat Supplementation

(a-b) Downregulated genes by P-80 supplementation present in empty vector (EV)- and *ech-6* RNAi-treated worms (G2) and exclusively present in empty vector (EV)-treated worms (G3) are primarily enriched in growth and development-associated processes. GO terms were considered significantly enriched for a modified Fisher’s Exact *p*-value < 0.05 (an EASE score).

**Table S1.** Lifespan Statistics

Summary of median lifespan and statistical analysis (*p*-values) for lifespan experiments displayed in Figs. 1b, c, Figs. 2a, b, Figs. 2e-h, Fig. 6e. Larval stage 4 (L4) is considered as day 0 of each lifespan assay. The median and *p*-values were calculated by a log-rank (Mantel-Cox) statistical test. *P*-values less than 0.05 are considered statistically significant, demonstrating that the two lifespan populations are different. Statistics of individual experiments are shown for each condition. The number of individuals scored, and independent experiments are shown. a: versus *ech-6* RNAi in N2; b: versus EV + 200 µM P-80; c: versus *ech-6* RNAi + 100 µM P-80 in N2.

**Table S2**. Statistical analysis of lipidomics

**Table S3**. Statistical analysis of RNAseq data

**Table S4.** Commonly increased and decreased lipids by *ech-6* RNAi, P-20 and P-80 supplementation, related to supplementary Figure S3.

**Table S5.** Fatty acid levels, related to Fig.1d and 1e.
